# Plasma Epstein-Barr viral DNA complements TNM classification of nasopharyngeal carcinoma in the era of intensity-modulated radiotherapy

**DOI:** 10.18632/oncotarget.6754

**Published:** 2015-12-24

**Authors:** Lu Zhang, Lin-Quan Tang, Qiu-Yan Chen, Huai Liu, Shan-Shan Guo, Li-Ting Liu, Ling Guo, Hao-Yuan Mo, Chong Zhao, Xiang Guo, Ka-Jia Cao, Chao-Nan Qian, Mu-Sheng Zeng, Jian-Yong Shao, Ying Sun, Jun Ma, Ming-Huang Hong, Hai-Qiang Mai

**Affiliations:** ^1^ Sun Yat-Sen University Cancer Center, State Key Laboratory of Oncology in South China, Collaborative Innovation Center for Cancer Medicine, Guangzhou, P. R. China; ^2^ Department of Nasopharyngeal Carcinoma, Sun Yat-Sen University Cancer Center, Guangzhou, P. R. China; ^3^ GCP Center, Sun Yat-Sen University Cancer Center, Guangzhou, P. R. China; ^4^ Department of Radiotherapy, Hunan Cancer Hospital, Changsha, P. R. China; ^5^ Department of Radiotherapy, The Affiliated Cancer Hospital of Xiangya School of Medicine, Central South University, Changsha, P. R. China; ^6^ Key Laboratory of Translational Radiation Oncology, Changsha, P. R. China; ^7^ Department of Molecular Diagnostics, Sun Yat-Sen University Cancer Center, Guangzhou, P. R. China; ^8^ Department of Radiation Oncology, Sun Yat-Sen University Cancer Center, Guangzhou, P. R. China

**Keywords:** nasopharyngeal carcinoma, Epstein-Barr viral DNA, TNM staging, intensity-modulated radiotherapy, prognosis

## Abstract

**Background:**

The objective of this study is to verify the prognostic value of pretreatment plasma Epstein-Barr viral deoxyribonucleic acid (pEBV DNA) levels in nasopharyngeal carcinoma (NPC) patients to complement TNM classification based on the application of the intensity-modulated radiotherapy (IMRT) technique.

**Methods:**

In total, 1467 patients staged at I–IVa–b (M0) and treated with IMRT were retrospectively analyzed at our cancer center from January 2007 to December 2010. Patient survival among different stages and EBV DNA levels were compared.

**Results:**

Outcome analyses of different stages and EBV DNA levels revealed that patients in stages II–III with low EBV DNA levels had similar survival as that of patients in stages IVa–b with low EBV DNA (5-yr overall survival (OS), 94.7% vs. 92.9% (*P* = 0.141), progression failure-free survival (PFS), 87.2% vs. 89.0% (*P* = 0.685), distant metastasis failure-free survival (DMFS), 93.5% vs. 92.4% (*P* = 0.394) and locoregional failure-free survival (LRFS), 93.8% vs. 96.3% (*P* = 0.523)). Conversely, patients in stages II–III with high EBV DNA had better survival than patients in stages IVa–b with high EBV DNA (5-yr OS, 82.7% vs. 71.7% (*P* = 0.001), PFS, 70.7% vs. 66.2% (*P* = 0.047), DMFS, 79.6% vs. 74.8% (*P* = 0.066) and LRFS, 89.3% vs. 87.6% (*P* = 0.425)) but poorer survival than patients in stages IVa–b with low EBV DNA (5-yr OS, 82.7% vs. 92.9% (*P* = 0.025), PFS, 70.7% vs. 89.0, (*P* < 0.001), DMFS, 79.6% vs. 92.4%, (*P* = 0.001), LRFS, 89.3% vs. 96.3%, (*P* = 0.022)).

**Conclusion:**

pEBV DNA is a strong prognostic factor for patients with NPC when complemented with TNM staging in the era of IMRT application.

## INTRODUCTION

Nasopharyngeal carcinoma (NPC) is a rare disease in Western countries, whereas it is endemic in Southern China [1]. According to NCCN guidelines, treatment for early (stage I) disease should be RT alone, though concurrent chemoradiotherapy (CCRT) with or without adjuvant chemotherapy is recommended for advanced (stages II–IVa–b) diseases. Currently, the TNM staging system is crucial for predicting the prognosis of NPC and guiding treatment management for different stages. However, as highly variable treatment outcomes have been reported in patients at the same stage [2, 3], reliable biomarkers that complement the present TNM staging system are very important for more accurate prognosis and more precise guidance of treatment decisions.

It has long been established that the pretreatment EBV DNA (pEBV DNA) level is associated with patient survival and can serve as a potential powerful biomarker for the prognosis of patients with NPC [4–16]. Indeed, a study by Leung et al. [4] showed that the pEBV DNA load is a prognostic factor in NPC independent of TNM staging, with the combined interpretation of EBV DNA and TNM staging data leading to changes in the risk definition of patient subsets. However, in that study, only 1 patient was treated by intensity-modulated radiotherapy (IMRT). With the advancement of technologies, modern irradiation for NPC should be IMRT with inverse RT planning, and it is not difficult to justify the implementation of IMRT as an excellent therapeutic intervention for improving local and regional control and patient survival [17–23]. Recently, a study conducted by Chen M et al. suggested that NPC patients with a high pEBV DNA level had worse disease-free survival (DFS) at only two years of follow-up time [24]. Thus, in the era of IMRT, it remains very important and necessary to demonstrate the clinical significance of pEBV DNA as a complement to TNM staging.

In this study, we report a large retrospective study involving 1467 patients with a median follow-up of 47 months after radiotherapy and offer more accurate, rigorous and practical information for clinical use on the basis of these data.

## RESULTS

### Patient characteristics

Among 1503 cases, a total of 1467 patients in stages I to IVa–b met the criteria and were enrolled in this large cohort retrospective clinical study. Table 1 listed the pretreatment characteristics of the 1467 NPC patients according to their levels of EBV DNA (a low EBV DNA group with EBV DNA < 4000 copies/ml and a high EBV DNA group with EBV DNA ≥ 4000 copies/ml). We observed no statistically significant differences in characteristics of age, sex, Eastern Cooperative Oncology Group (ECOG) and World Health Organization (WHO) type between the high and low EBV DNA groups, except for the T classification, N classification, overall stage and mode of treatment. The median follow-up time was 47 months (range, 1–103 months). Among these patients, 98 (6.7%) developed local and/or regional recurrences, 179 (12.2%) developed distant metastases, and 21 (1.4%) showed both local/regional and distant metastases. A total of 149 (10.2%) deaths were recorded at the date of the last follow-up.

**Table 1:** Patient and disease characteristics

Parameter	EBV DNA ≥ 4000 copies/ml		EBV DNA < 4000 copies/ml		*P* value
	No. of patients	%	No. of patients	%	
	648		819		
**Age (years)**					0.068
Range	11–96		11–94		
Median	46		45		
**Sex**					0.132
Male	474	73.1	568	69.4	
Female	174	26.9	251	30.6	
**ECOG**					0.660
≤ 1	645	99.5	817	99.8	
> 1	3	0.5	2	0.2	
**WHO classification**					0.342
II	29	4.5	46	5.6	
III	619	95.5	773	94.4	
**T classification**					< 0.001
T1–2	125	19.3	282	34.4	
T3–4	523	80.7	537	65.6	
**N classification**					< 0.001
N0–1	253	39.0	541	66.1	
N2–3	395	61.0	278	33.9	
Overall stage*					< 0.001
I	3	0.5	57	7.0	
II	30	4.6	143	17.5	
III	341	52.6	452	55.2	
IVa–b	274	42.3	167	20.4	
**Treatment strategy**					< 0.001
CRT^¶^	547	84.4	578	70.6	
RT^#^	101	15.6	241	29.4	
**Follow-up time, months**					
Median	44		48		
Range	1–85		1–103		0.002

Abbreviations: WHO, World Health Organization; ECOG, Eastern Cooperative Oncology Group; 2D-RT, two-dimensional radiotherapy; IMRT, intensity-modulated radiotherapy; DM, distant metastasis;

¶ CRT, chemoradiotherapy with or without sequential chemotherapy (induction or adjuvant).

# RT, radiotherapy with or without sequential chemotherapy (induction or adjuvant).

* The 7th AJCC/UICC staging system.

We determined the cut-off value of pEBV DNA concentration for OS by a receiver operating characteristic (ROC) curve and then obtained a value of 4340 copies/ml, similar to previous studies [10]. Because OS is the most commonly used endpoint for patient outcome in evaluating potential prognostic factors and the other cut-off points for PFS, DMFS and LRFS in our study were also close to the OS value, we selected 4000 copies/ml as the pEBV DNA cut-off point to define high and low EBV DNA levels for the overall endpoints.

### Prognostic value of TNM staging in NPC patients

**Figure 1:**
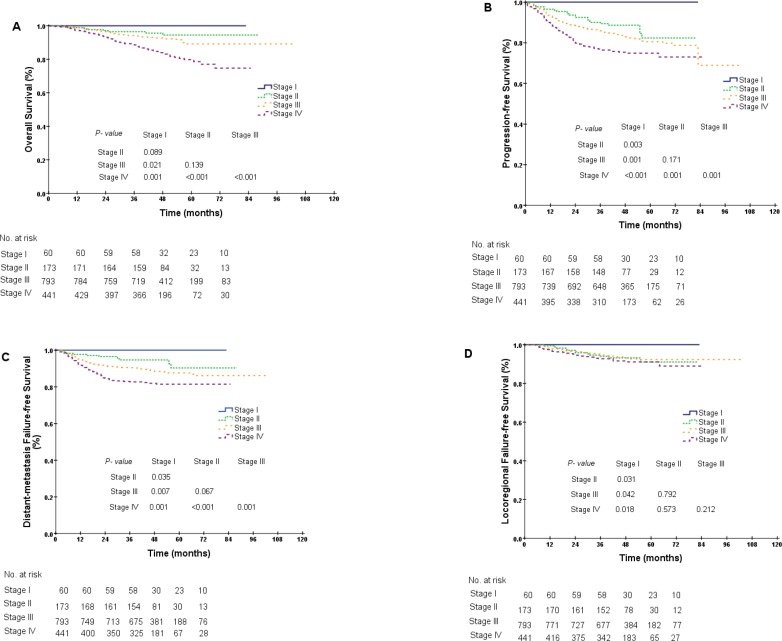
Kaplan-Meier curves for overall survival (**A**), progression-free survival (**B**), distant metastasis failure-free survival (**C**) and locoregional failure-free survival (**D**) in NPC patients at different stages.

**Figure 2:**
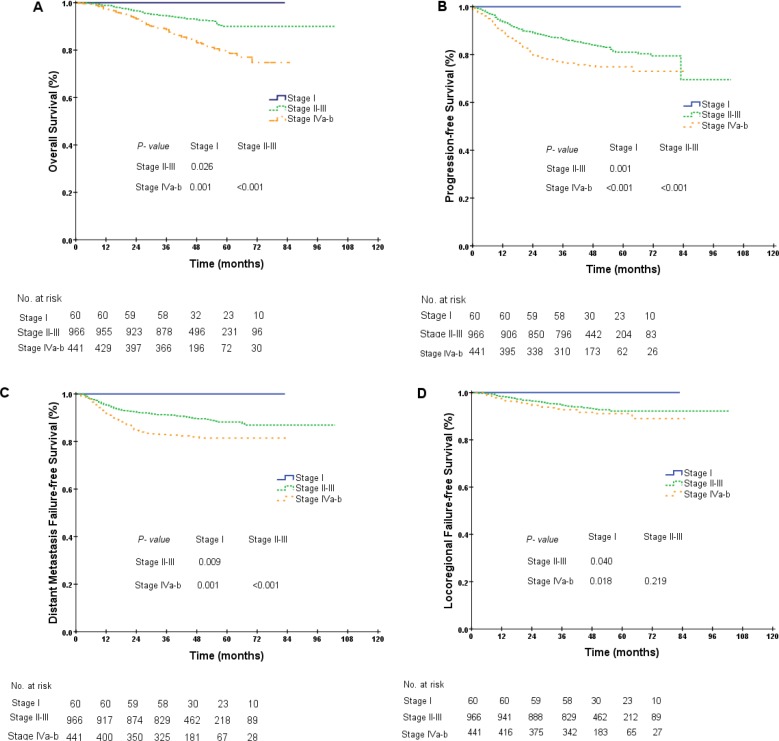
Kaplan-Meier curves for overall survival (**A**), progression-free survival (**B**), distant metastasis failure-free survival (**C**) and locoregional failure-free survival (**D**) in NPC patients of different groups (group 1, stage I; group 2, stages II–III; group 3, stages IVa–b).

As displayed in the Kaplan-Meier plot (Figure 1A), the 5-year OS difference between stage I and stage II was marginal (100.0% vs. 94.5%, *P* = 0.089), the difference between stage II and stage III was not significant (94.5% vs. 89.2%, *P* = 0.139), and the difference between stage III and stages IVa–b was significant (89.2% vs. 79.8%, *P* < 0.001). We repeated the Kaplan-Meier plot for PFS, DMFS and LRFS and found that the 5-year PFS was 100.0%, 82.3%, 80.5% and 74.9% among patients with stages I, II, III and IVa–b, respectively. The differences between adjacent stages were significant, except between stages II and III (stage II vs. stage III, 82.3% vs. 80.5%, *P* = 0.171, Figure 1B). Similar results were also obtained for the 5-year DMFS and LRFS between stages II and III (DMFS, 90.3% vs. 87.6%, *P* = 0.067, LRFS, 91.1% vs. 92.3%, *P* = 0.792, Figure 1C–1D). As the differences in survival probabilities between stage II and stage III were not all significant, we then segregated patients into three different groups: (1) group 1: stage I; (2) group 2: stages II–III; and (3) group 3: stages IVa–b. The rationale for this segregation did take into account the different treatment strategies recommended in NCCN guidelines (version 2.2014). After segregation, the comparisons for the 5-year survival probabilities between stage I and stages II–III were all significantly different (OS, 100.0% vs. 90.0%, *P* = 0.026, PFS, 100.0% vs. 81.0%, *P* = 0.001, DMFS, 100.0% vs. 88.2%, *P* = 0.009, LRFS, 100.0% vs. 92.1%, *P* = 0.040, Figure 2A–2D). Furthermore, the differences in OS, PFS and DMFS between stages II–III and stages IVa–b were also significant, except for the 5-year LRFS (OS, 90.0% vs. 79.8%, *P* < 0.001, PFS, 81.0% vs. 74.9%, *P* < 0.001, DMFS, 88.2% vs. 81.5%, *P* < 0.001, LRFS, 92.1% vs. 91.1%, *P* = 0.219, Figure 2A–2D).

### Prognostic value of pEBV DNA in the entire NPC patient cohort

**Figure 3:**
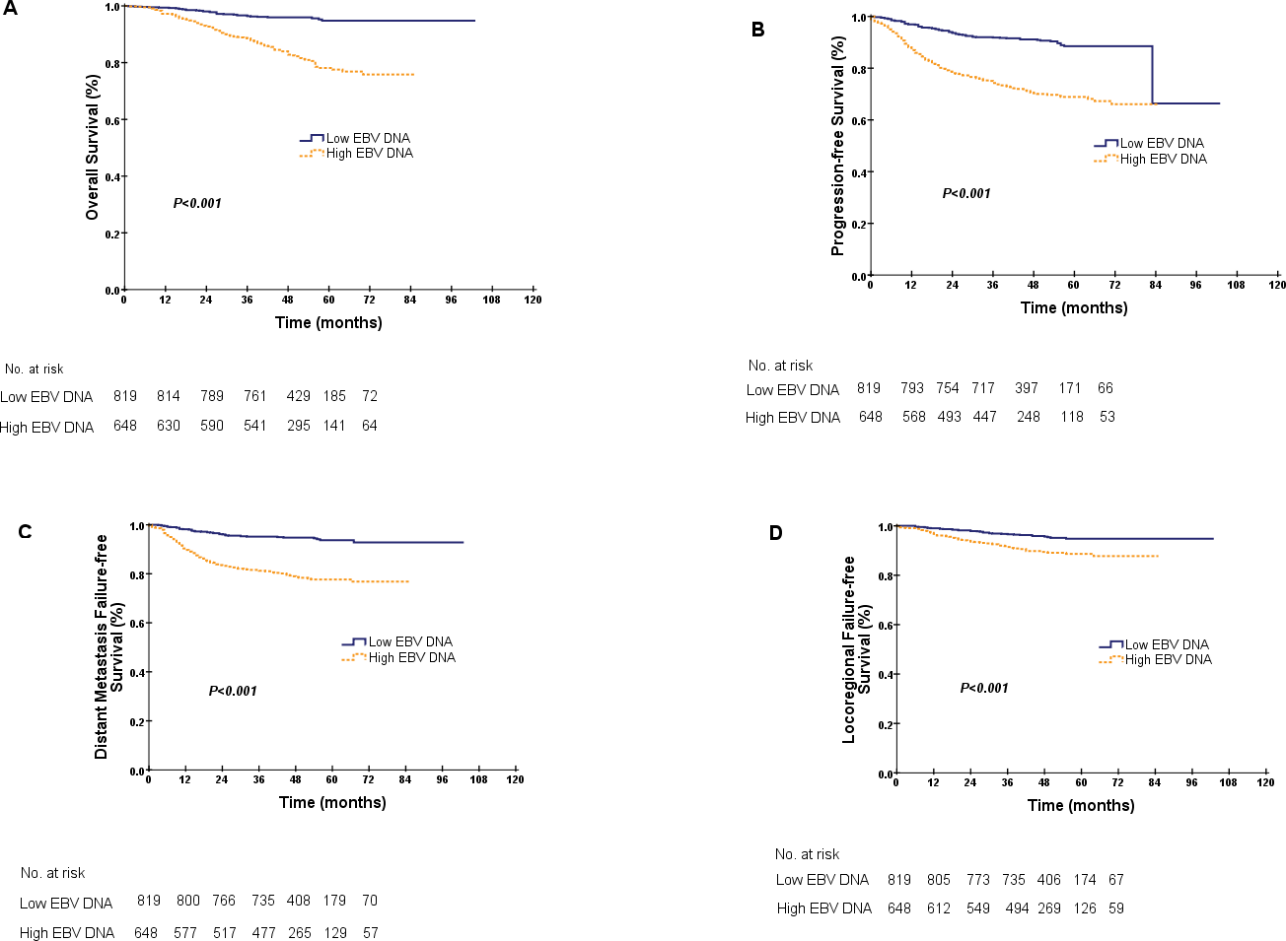
Kaplan-Meier curves for overall survival (**A**), progression-free survival (**B**), distant metastasis failure-free survival (**C**) and locoregional failure-free survival (**D**) for the entire cohort according to EBV DNA levels. A low EBV DNA denotes an EBV DNA concentration < 4000 copies/ml, and a high EBV DNA denotes an EBV DNA concentration ≥ 4000 copies/ml.

The patients were stratified into low EBV DNA and high EBV DNA groups according to their EBV DNA levels (EBV DNA < 4000 copies/ml and EBV DNA ≥ 4000 copies/ml). Kaplan-Meier estimates showed that the difference in OS between these EBV DNA levels was significant, and the 5-year OS values for the low and high EBV DNA groups were 94.8% and 78.1%, respectively (*P* < 0.001, Figure 3A). Analyses of PFS, DMFS and LRFS between the low and high EBV DNA levels were performed, with the same conclusions (PFS, 88.5% vs. 68.9%, DMFS, 93.7% vs. 77.7%, LRFS, 94.8% vs. 88.6%, with all *P* < 0.001, Figure 3B–3D).

### Prognostic value of pEBV DNA complemented by TNM staging

Patient survivals between the different subgroups after the introduction of pEBV DNA are listed in Table 2. As indicated, the patients at stages II–III with low EBV DNA levels had similar survival as patients at stages IVa–b with low EBV DNA (5-yr OS, 94.7% vs. 92.9% (*P* = 0.141), PFS, 87.2% vs. 89.0% (*P* = 0.685), DMFS, 93.5% vs. 92.4% (*P* = 0.394) and LRFS, 93.8% vs. 96.3% (*P* = 0.523)). However, patients at stages II–III with high EBV DNA levels had better survival than patients at stages IVa–b with high EBV DNA (5-yr OS, 82.7% vs. 71.7% (*P* = 0.001), PFS, 70.7% vs. 66.2% (*P* = 0.047), DMFS, 79.6% vs. 74.8% (*P* = 0.066) and LRFS, 89.3% vs. 87.6% (*P* = 0.425)) but poorer survival than patients at stages IVa–b with low EBV DNA (5-yr OS, 82.7% vs. 92.9% (*P* = 0.025), PFS, 70.7% vs. 89.0, (*P* < 0.001), DMFS, 79.6% vs. 92.4%, (*P* = 0.001), LRFS, 89.3% vs. 96.3%, (*P* = 0.022)). Furthermore, survival comparisons between the high and low EBV DNA groups at stages II–III or stages IVa–b were significantly different, as shown in S. table 1 (Supplementary Material 1).

**Table 2:** Survival comparisons between among subgroups

Stratification	OS		PFS		DMFS		LRFS	
	5 year (%)	*P* value	5 year (%)	*P* value	5 year (%)	*P* value	5 year (%)	*P* value
I vs. II–III low EBV DNA	100% vs. 94.7%	0.106	100% vs.87.2%	0.013	100% vs. 93.5%	0.056	100% vs. 93.8%	0.080
I vs. IVa–b low EBV DNA	100% vs. 92.9%	0.041	100% vs. 89.0%	0.010	100% vs. 92.4%	0.033	100% vs. 96.3%	0.133
I vs. II–IVa–b low EBV DNA	100% vs. 94.3%	0.094	100% vs. 89.2%	0.015	100% vs. 93.2%	0.055	100% vs. 94.4%	0.097
II–III low EBV DNA vs. IVa–b low EBV DNA	94.7% vs. 92.9%	0.141	87.2% vs. 89.0%	0.685	93.5% vs. 92.4%	0.394	93.8% vs. 96.3%	0.523
II–III high EBV DNA vs. IVa–b low EBV DNA	82.7% vs. 92.9%	0.025	70.7% vs. 89.0%	< 0.001	79.6% vs. 92.4%	0.001	89.3% vs. 96.3%	0.022
II–III high EBV DNA vs. IVa–b high EBV DNA	82.7% vs. 71.7%	0.001	70.7% vs. 66.2%	0.047	79.6% vs. 74.8%	0.066	89.3% vs. 87.6%	0.425

Abbreviations: OS, overall survival; PFS, progression-free survival; DMFS, distant failure-free survival; LRFS, locoregional failure-free survival.

All the 5 year survival rates were calculated using the Kaplan-Meier method.

EBV DNA level was defined as high EBV DNA with concentrations greater than or equal to 4000 copies/ml and low EBV DNA with concentrations smaller than 4000 copies/ml.

*P* values achieved between different subgroups.

### COX multivariate analysis

COX multivariate proportional hazards model analyses revealed the plasma EBV DNA level to be a more important prognostic factor than any other characteristic such as age, sex, overall stage, or treatment strategy (Table 3). As shown in Table 3, EBV DNA levels were significantly associated with OS (hazard ratio [HR] = 3.435, 95% CI, 2.317–5.093, *P* < 0.001), PFS (HR = 3.304, 95% CI, 2.482–4.398, *P* < 0.001), DMFS (HR = 3.549, 95% CI, 2.491–5.055, *P* < 0.001) and LRFS (HR = 2.510, 95% CI, 1.615–3.901, *P* < 0.001).

**Table 3:** Result of multivariate COX proportional hazards model analysis for the whole cohort

Parameter	OS		PFS		DMFS		LRFS	
	HR (95% CI)	*P* value*	HR (95% CI)	*P* value	HR (95% CI)	*P* value	HR (95% CI)	*P* value
**Age:** ≤ 45y vs. > 45y	1.503 (1.079, 2.093)	0.016	1.055 (0.824, 1.351)	0.669	1.140 (0.847, 1.535)	0.388	0.809 (0.541, 1.209)	0.301
**Sex:** female vs. male	1.497 (0.995, 2.250)	0.053	1.224 (0.920, 1.628)	0.166	1.476 (1.025, 2.124)	0.036	1.077 (0.690, 1.682)	0.744
**Stage classification:** I–IVa–b	1.801 (1.369, 2.370)	< 0.001	1.416 (1.165, 1.721)	< 0.001	1.454 (1.142, 1.851)	0.002	1.245 (0.921, 1.683)	0.154
**Pretreatment EBV DNA:** < 4000 vs. ≥ 4000	3.435 (2.317, 5.093)	< 0.001	3.304 (2.482, 4.398)	< 0.001	3.549 (2.491, 5.055)	< 0.001	2.510 (1.615, 3.901)	< 0.001
**Strategy:** RT^#^ vs. CRT^¶^	0.723 (0.477, 1.097)	0.128	0.628 (0.464, 0.851)	0.003	0.776 (0.529, 1.138)	0.195	0.533 (0.332, 0.854)	0.009

Abbreviations: CI, confidence interval; WHO, world health organization; OS, overall survival; PFS, progression-free survival; DMFS, distant metastasis failure-free survival; LRFS, locoregional failure-free survival.

* The Wald test was used to test *P* values.

# RT, radiotherapy with or without sequential chemotherapy (induction or adjuvant).

¶ CRT, chemoradiotherapy with or without sequential chemotherapy (induction or adjuvant).

## DISCUSSION

To the best of our knowledge, our current study utilizes the largest sample size to verify the biomarker of EBV DNA as an independent prognostic factor for NPC patients, regardless of TNM staging, and also provides useful data for the stratification of patients into different risk groups for treatment selection on the basis of the application of IMRT. The data we obtained suggested that a high level of EBV DNA is an independent adverse prognostic factor for patient survival, in accordance with previous studies [4, 5, 10, 15]. Our data also underlined the importance of re-classifying patients into four subgroups when combining the EBV DNA level with the TNM staging system.

In our study, the TNM staging system failed to distinguish between stage II and stage III patients in predicting survival. Thus, it appears reasonable to combine stages II and III to form a new risk group with unfavorable survival compared with that of stage I patients but with favorable survival compared with that of stages IVa–b patients. Furthermore, after the introduction of EBV DNA to the issue of risk stratification, patients in stages II–III with high EBV DNA showed a poorer survival than patients in stages IVa–b with low EBV DNA and a better survival than patients in stages IVa–b with high EBV DNA. Patients with low EBV DNA levels between stages II–III and stages IVa–b had similar survival, the combined subgroup (stages II–IVa–b with low EBV DNA) showed worse survival compared with stage I patients. Based on our data presented above, patients can be segregated into four different risk groups: (1) very low risk, stage I; (2) low risk, stages II–IVa–b with low EBV DNA; (3) intermediate risk, stages II–III with high EBV DNA; (4) high risk, stages IVa–b with high EBV DNA. In accordance with the current NCCN guidelines, RT alone is sufficient for stage I, whereas CCRT with or without adjuvant chemotherapy may be needed for stages II to IVa–b. However, our current study offers a novel change in the risk definition for NPC patients, which may guide design of future individualized clinical trial. Based on our data, for low risk patients (stages II–IVa–b with low EBV DNA), IMRT with concurrent chemotherapy alone may be sufficient. Nonetheless, the use of concurrent chemoradiotherapy with two or three cycles of cisplatin chemotherapy still needs to be decided, as reported previously [25, 26]. For patients of the intermediate risk group (stages II–III with high EBV DNA), though the uncertain role of adjuvant chemotherapy [27–29], more intense treatment strategy such as CCRT plus adjuvant chemotherapy may be required. For patients in the high-risk group (stages IVa–b with high EBV DNA), the addition of immune therapy or targeted therapy may be necessary.

Our study reveals once again that EBV DNA is attractive and useful in medical practices. Two previous studies have suggested that EBV DNA has better prognostic value when combined with the TNM staging system. First, Leung and colleagues [16] performed a prospective study to assess the prognostic effect of pEBV DNA in patients with early-stage NPC, and the results revealed that the probability of distant failure was significantly higher in patients with high EBV DNA levels than in patients with low EBV DNA levels (*P* = 0.0001). The authors concluded that the pretherapy EBV DNA level could identify a poor risk group with a probability of distant failure similar to patients with advanced-stage disease. Soon thereafter, Leung et al. [4] again reported the pretherapy EBV DNA load as an independent prognostic factor of the TNM staging system in NPC, concluding that EBV DNA could lead to an altered risk definition of patient subsets when combined with TNM staging, with improved risk discrimination in early-stage disease. However, one point we should highlight is that no patients in the first study and only one patient in the second study received IMRT.

Although this study has many clinical implications, we should be clear that it is a retrospective study with its own limitations. First, our study was conducted in a single institution. Second, longer follow-up may be needed to accurately reflect cancer recurrence or death. Third, more studies of EBV DNA levels complemented by TNM staging in the era of IMRT, especially prospective studies, should be performed in the future.

## CONCLUSION

In summary, this study confirmed that pEBV DNA is a strong prognostic factor for patients with NPC when complemented with TNM staging in the era of IMRT application. It is advised that patients be segregated into four risk groups and suggested that different intense treatment protocols may be considered. The role of pEBV DNA in guiding individualized clinical trial is needed to be evaluated.

## PATIENTS AND METHODS

### Subjects

This work is a retrospective study involving a total of 1503 patients presenting to the institute with previously untreated NPC from January 2007 to December 2010. Inclusion criteria included the following: (1) patients with biopsy-proven WHO type II or III; (2) ECOG of 0 to 2; (3) receiving IMRT treatment; (4) adequate hematologic, renal, and hepatic function. Exclusion criteria were as follows: (1) history of previous anticancer therapy, (2) pregnancy or lactation; (3) a history of previous or synchronous malignant tumors; (4) the presence of a primary distant metastasis; (5) patients lost during follow-up or no available EBV DNA data. The routine staging workup prior to treatment consisted of a detailed history, physical examination of the head and neck, nasopharyngeal endoscopy, magnetic resonance imaging (MRI) and/or computed tomography (CT), complete blood counts, biochemical profile, pretreatment plasma testing for EBV DNA level, chest X-ray, abdominal ultrasound, whole-body bone scan, and dental evaluation. As the cancer stages of some of the patients were staged according to the 6th edition of the International Union Against Cancer (UICC) and the American Joint Committee on Cancer (AJCC) staging-system manual, we reclassified patient stages using the 2010 UICC/AJCC staging system (7th edition).

### IMRT treatment and chemotherapy treatment

All patients included in this study were treated with IMRT technology. Similar to the references RTOG 0615 [18] and RTOG 0225 [17], we delineated the target volumes using the institutional treatment protocol, as previously described [30–32], in accordance with the International Commission on Radiation Units and Measurements reports 50 and 62.

Of the 1234 patients with stages III to IVa–b cancer, approximately 1048 (84.9%) received chemoradiotherapy with or without neoadjuvant or adjuvant chemotherapy using various regimens of cytotoxic drugs. The selection of chemotherapy regimen and sequential chemotherapy was based on specific circumstances and the discretion of the doctors, but all chemotherapies were platinum based. Of the 233 patients with stage I to II cancer, all 60 stage I patients and 81 stage II patients received RT alone. A total of 14 (6.0%) patients at stage II received induction chemotherapy sequential to RT, and 78 (33.5%) received CCRT with or without neoadjuvant or adjuvant chemotherapy.

### Patient follow-up

All patients were evaluated at the end of the irradiation treatment. In addition, the patients were required to be followed up every 3 months during the first 3 years, every 6 months during the next two years, and then annually thereafter. At each follow-up visit, endoscopy, physical examination, basic chemical profiles chest X-ray, abdominal ultrasound and head and neck MRI were performed every 6 months. Bone scan and CT of the chest or abdomen and even PET/CT were performed when clinically indicated.

### Data analysis

The primary endpoint of our study was OS, defined as the time interval from the commencement of treatment until death from any cause or when censored at the date of the last follow-up. The secondary endpoints included PFS, DMFS and LRFS. PFS was defined as the time interval from the commencement of treatment to the date of the first observation of recurrence, death or censored at the date of the last follow-up. DMFS was defined as the time interval from the commencement of treatment to the date of the first observation of distant lesion or censored at the date of the last follow-up. LRFS was defined as the time interval from the commencement of treatment to the date of the first observation of local and/or regional failure or censored at the date of the last follow-up. In the current study, patient survival was calculated by Kaplan-Meier curves, and the survival probability between different groups was compared using the log rank test. The characteristics between subgroups were compared using Chi-square and Wilcoxon rank sum tests. Then, a COX proportional hazards model was used to examine the association of various prognostic factors, including the levels of pEBV DNA, age, sex, overall stage, and treatment strategy. All statistical tests were two-sided, and *P* values of < 0.05 were considered statistically significant. The statistical analyses were performed using SPSS version 17.0 (SPSS, Chicago, IL, USA).

## SUPPLEMENTARY MATERIAL TABLE



## References

[1] Shin HR, Masuyer E, Ferlay J, Curado MP (2010). Cancer in Asia - Incidence rates based on data in cancer incidence in five continents IX (1998–2002). Asian Pac J Cancer Prev.

[2] Mao YP, Xie FY, Liu LZ, Sun Y, Li L, Tang LL, Liao XB, Xu HY, Chen L, Lai SZ, Lin AH, Liu MZ, Ma J (2009). Re-evaluation of 6th edition of AJCC staging system for nasopharyngeal carcinoma and proposed improvement based on magnetic resonance imaging. Int J Radiat Oncol Biol Phys.

[3] Wang HY, Sun BY, Zhu ZH, Chang ET, To KF, Hwang JS, Jiang H, Kam MK, Chen G, Cheah SL, Lee M, Liu ZW, Chen J (2011). Eight-signature classifier for prediction of nasopharyngeal [corrected] carcinoma survival. J Clin Oncol.

[4] Leung SF, Zee B, Ma BB, Hui EP, Mo F, Lai M, Chan KC, Chan LY, Kwan WH, Lo YM, Chan AT (2006). Plasma Epstein-Barr viral deoxyribonucleic acid quantitation complements tumor-node-metastasis staging prognostication in nasopharyngeal carcinoma. J Clin Oncol.

[5] Lin JC, Wang WY, Chen KY, Wei YH, Liang WM, Jan JS, Jiang RS (2004). Quantification of plasma Epstein-Barr virus DNA in patients with advanced nasopharyngeal carcinoma. N Engl J Med.

[6] Tang LQ, Chen QY, Guo SS, Chen WH, Li CF, Zhang L, Lai XP, He Y, Xu YX, Hu DP, Wen SH, Peng YT, Liu H (2014). The impact of plasma Epstein-Barr virus DNA, fibrinogen on nasopharyngeal carcinoma prognosis: an observational study. Br J Cancer.

[7] Hui EP, Ma BB, Chan KC, Chan CM, Wong CS, To KF, Chan AW, Tung SY, Ng WT, Cheng AC, Lee VH, Chan SL, Loong HH (2015). Clinical utility of plasma Epstein-Barr virus DNA, ERCC1 single nucleotide polymorphism in nasopharyngeal carcinoma. Cancer.

[8] Leung SF, Chan KC, Ma BB, Hui EP, Mo F, Chow KC, Leung L, Chu KW, Zee B, Lo YM, Chan AT (2014). Plasma Epstein-Barr viral DNA load at midpoint of radiotherapy course predicts outcome in advanced-stage nasopharyngeal carcinoma. Ann Oncol.

[9] Zhang W, Chen Y, Chen L, Guo R, Zhou G, Tang L, Mao Y, Li W, Liu X, Du X, Sun Y, Ma J (2015). The clinical utility of plasma Epstein-Barr virus DNA assays in nasopharyngeal carcinoma: the dawn of a new era?: a systematic review and meta-analysis of 7836 cases. Medicine (Baltimore).

[10] Chan AT, Lo YM, Zee B, Chan LY, Ma BB, Leung SF, Mo F, Lai M, Ho S, Huang DP, Johnson PJ (2002). Plasma Epstein-Barr virus DNA and residual disease after radiotherapy for undifferentiated nasopharyngeal carcinoma. J Natl Cancer Inst.

[11] Lin JC, Chen KY, Wang WY, Jan JS, Liang WM, Tsai CS, Wei YH (2001). Detection of Epstein-Barr virus DNA in the peripheral-blood cells of patients with nasopharyngeal carcinoma: relationship to distant metastasis and survival. J Clin Oncol.

[12] Lin JC, Wang WY, Liang WM, Chou HY, Jan JS, Jiang RS, Wang JY, Twu CW, Liang KL, Chao J, Shen WC (2007). Long-term prognostic effects of plasma epstein-barr virus DNA by minor groove binder-probe real-time quantitative PCR on nasopharyngeal carcinoma patients receiving concurrent chemoradiotherapy. Int J Radiat Oncol Biol Phys.

[13] Bortolin MT, Pratesi C, Dolcetti R, Bidoli E, Vaccher E, Zanussi S, Tedeschi R, De Paoli P (2006). Clinical value of Epstein-Barr virus DNA levels in peripheral blood samples of Italian patients with undifferentiated carcinoma of nasopharyngeal type. Cancer Lett.

[14] Mutirangura A, Pornthanakasem W, Theamboonlers A, Sriuranpong V, Lertsanguansinchi P, Yenrudi S, Voravud N, Supiyaphun P, Poovorawan Y (1998). Epstein-Barr viral DNA in serum of patients with nasopharyngeal carcinoma. Clin Cancer Res.

[15] Lo YM, Chan AT, Chan LY, Leung SF, Lam CW, Huang DP, Johnson PJ (2000). Molecular prognostication of nasopharyngeal carcinoma by quantitative analysis of circulating Epstein-Barr virus DNA. Cancer Res.

[16] Leung SF, Chan AT, Zee B, Ma B, Chan LY, Johnson PJ, Lo YM (2003). Pretherapy quantitative measurement of circulating Epstein-Barr virus DNA is predictive of posttherapy distant failure in patients with early-stage nasopharyngeal carcinoma of undifferentiated type. Cancer.

[17] Lee N, Harris J, Garden AS, Straube W, Glisson B, Xia P, Bosch W, Morrison WH, Quivey J, Thorstad W, Jones C, Ang KK (2009). Intensity-modulated radiation therapy with or without chemotherapy for nasopharyngeal carcinoma: radiation therapy oncology group phase II trial 0225. J Clin Oncol.

[18] Lee NY, Zhang Q, Pfister DG, Kim J, Garden AS, Mechalakos J, Hu K, Le QT, Colevas AD, Glisson BS, Chan AT, Ang KK (2012). Addition of bevacizumab to standard chemoradiation for locoregionally advanced nasopharyngeal carcinoma (RTOG 0615): a phase 2 multi-institutional trial. Lancet Oncol.

[19] Zeng L, Tian YM, Sun XM, Chen CY, Han F, Xiao WW, Deng XW, Lu TX (2014). Late toxicities after intensity-modulated radiotherapy for nasopharyngeal carcinoma: patient and treatment-related risk factors. Br J Cancer.

[20] Lin S, Pan J, Han L, Guo Q, Hu C, Zong J, Zhang X, Lu JJ (2014). Update report of nasopharyngeal carcinoma treated with reduced-volume intensity-modulated radiation therapy and hypothesis of the optimal margin. Radiother Oncol.

[21] Wu F, Wang R, Lu H, Wei B, Feng G, Li G, Liu M, Yan H, Zhu J, Zhang Y, Hu K (2014). Concurrent chemoradiotherapy in locoregionally advanced nasopharyngeal carcinoma: treatment outcomes of a prospective, multicentric clinical study. Radiother Oncol.

[22] Ng WT, Lee MC, Chang AT, Chan OS, Chan LL, Cheung FY, Hung WM, Chan CC, Lee AW (2014). The impact of dosimetric inadequacy on treatment outcome of nasopharyngeal carcinoma with IMRT. Oral Oncol.

[23] Yi J, Huang X, Gao L, Luo J, Zhang S, Wang K, Qu Y, Xiao J, Xu G (2014). Intensity-modulated radiotherapy with simultaneous integrated boost for locoregionally advanced nasopharyngeal carcinoma. Radiat Oncol.

[24] Chen M, Yin L (2015). Impact of plasma Epstein-Barr virus-DNA and tumor volume on prognosis of locally advanced nasopharyngeal carcinoma. Biomed Res Int.

[25] Lee AW, Tung SY, Ngan RK, Chappell R, Chua DT, Lu TX, Siu L, Tan T, Chan LK, Ng WT, Leung TW, Fu YT, Au GK (2011). Factors contributing to the efficacy of concurrent-adjuvant chemotherapy for locoregionally advanced nasopharyngeal carcinoma: combined analyses of NPC-9901 and NPC-9902 Trials. Eur J Cancer.

[26] Loong HH, Chan AT (2014). Controversies in the systemic treatment of nasopharyngeal carcinoma. Oral Oncol.

[27] Langendijk JA, Leemans CR, Buter J, Berkhof J, Slotman BJ (2004). The additional value of chemotherapy to radiotherapy in locally advanced nasopharyngeal carcinoma: a meta-analysis of the published literature. J Clin Oncol.

[28] Huncharek M, Kupelnick B (2002). Combined chemoradiation versus radiation therapy alone in locally advanced nasopharyngeal carcinoma: results of a meta-analysis of 1,528 patients from six randomized trials. Am J Clin Oncol.

[29] Chen L, Hu CS, Chen XZ, Hu GQ, Cheng ZB, Sun Y, Li WX, Chen YY, Xie FY, Liang SB, Chen Y, Xu TT, Li B (2012). Concurrent chemoradiotherapy plus adjuvant chemotherapy versus concurrent chemoradiotherapy alone in patients with locoregionally advanced nasopharyngeal carcinoma: a phase 3 multicentre randomised controlled trial. Lancet Oncol.

[30] Lai SZ, Li WF, Chen L, Luo W, Chen YY, Liu LZ, Sun Y, Lin AH, Liu MZ, Ma J (2011). How does intensity-modulated radiotherapy versus conventional two-dimensional radiotherapy influence the treatment results in nasopharyngeal carcinoma patients?. Int J Radiat Oncol Biol Phys.

[31] Xiao WW, Huang SM, Han F, Wu SX, Lu LX, Lin CG, Deng XW, Lu TX, Cui NJ, Zhao C (2011). Local control, survival, and late toxicities of locally advanced nasopharyngeal carcinoma treated by simultaneous modulated accelerated radiotherapy combined with cisplatin concurrent chemotherapy: long-term results of a phase 2 study. Cancer.

[32] Su SF, Han F, Zhao C, Chen CY, Xiao WW, Li JX, Lu TX (2012). Long-term outcomes of early-stage nasopharyngeal carcinoma patients treated with intensity-modulated radiotherapy alone. Int J Radiat Oncol Biol Phys.

