# Supplementary material for: Plasma Epstein-Barr viral DNA complements TNM classification of nasopharyngeal carcinoma in the era of intensity-modulated radiotherapy
Source: Oncotarget. 2015 Dec 24;7(5):6221–30. doi: 10.18632/oncotarget.6754 (PMC4868751; doi:10.18632/oncotarget.6754)
Supplement: Supplementary file 1 [file oncotarget-07-6221-s001.pdf]

Plasma Epstein-Barr viral DNA complements TNM classification of nasopharyngeal carcinoma in the era of intensity-modulated radiotherapy

Supplementary Materials

Supplementary Table S1: Survival analysis comparisons of EBV DNA within different subgroups in the whole population

Stage and EBV DNA level	No. of Patients	OS		PFS		DMFS		LRFS	
		Events	5 year	Events	5 year	Events	5 year	Events	5 year
**I**	60								
High EBV DNA	3 (5.0%)	0 (0.0%)	100.0%	0 (0.0%)	100.0%	0 (0.0%)	100.0%	0 (0.0%)	100.0%
Low EBV DNA	57 (95.0%)	0 (0.0%)	100.0%	0 (0.0%)	100.0%	0 (0.0%)	100.0%	0 (0.0%)	100.0%
P value			–		–		–		–
**II**	173								
High EBV DNA	30 (17.3%)	3 (10.0%)	83.4%	8 (26.7%)	72.5%	4 (13.3%)	86.5%	4 (13.3)	85.6%
Low EBV DNA	143 (82.7%)	5 (3.5%)	96.5%	14 (9.8%)	84.4%	7 (4.9%)	91.1%	8 (5.6)	92.2%
P value			0.113		0.008		0.075		0.110
**III**	793								
High EBV DNA	341 (43.0%)	47 (13.8%)	82.5%	91 (26.8%)	70.7%	65 (19.1%)	79.1%	30 (8.8%)	89.7%
Low EBV DNA	452 (57.0%)	19 (4.2%)	94.3%	47 (10.4%)	87.8%	26 (5.8%)	93.9%	21 (4.6%)	94.2%
P value			< 0.001		< 0.001		< 0.001		0.010
**II–III**	966								
High EBV DNA	371	50 (13.5%)	82.7%	99 (26.8%)	70.7%	68 (18.4%)	79.6%	34 (9.2%)	89.3%
Low EBV DNA	595	24 (4.0%)	94.7%	61 (10.3%)	87.2%	33 (5.5%)	93.5%	29 (4.9%)	93.8%
P value			< 0.001		< 0.001		< 0.001		< 0.001
**IVa–b**	441								
High EBV DNA	274 (62.1%)	64 (23.4%)	71.7%	89 (32.5%)	66.2%	65 (23.7%)	74.8%	29 (10.6%)	87.6%
Low EBV DNA	167 (37.9%)	11 (6.6%)	92.9%	18 (10.8%0	89.0%	12 (7.2%)	92.4%	6 (3.6%)	96.3%
P value			< 0.001		< 0.001		< 0.001		0.006

Abbreviations: OS, overall survival; PFS, progression-free survival; DMFS, distant failure-free survival; LRFS, locoregional failure-free survival.
All the 5-year survival rates were calculated using the Kaplan-Meier method. EBV DNA level was defined as high EBV DNA with concentrations greater than or equal to 4000 copies/ml and low EBV DNA with concentrations smaller than 4000 copies/ml.
P values achieved within different subgroups for survival with high EBV DNA level vs. low EBV DNA level.
